# Genetic variation in a grapevine progeny (*Vitis vinifera* L. cvs Grenache×Syrah) reveals inconsistencies between maintenance of daytime leaf water potential and response of transpiration rate under drought

**DOI:** 10.1093/jxb/eru228

**Published:** 2014-06-13

**Authors:** Aude Coupel-Ledru, Éric Lebon, Angélique Christophe, Agnès Doligez, Llorenç Cabrera-Bosquet, Philippe Péchier, Philippe Hamard, Patrice This, Thierry Simonneau

**Affiliations:** ^1^INRA Laboratoire d’Ecophysiologie des Plantes sous Stress Environnementaux, Place Viala, F-34060 Montpellier Cedex 1, France; ^2^INRA UMR AGAP, Place Viala, F-34060 Montpellier Cedex 1, France

**Keywords:** Anisohydric, drought, high-throughput phenotyping, hydraulic conductance, isohydric, leaf water potential, QTL, transpiration rate, *Vitis vinifera* L.

## Abstract

In the face of water stress, plants evolved with different abilities to limit the decrease in leaf water potential, notably in the daytime (Ψ_M_). So-called isohydric species efficiently maintain high Ψ_M_, whereas anisohydric species cannot prevent Ψ_M_ from dropping as soil water deficit develops. The genetic and physiological origins of these differences in (an)isohydric behaviours remain to be clarified. This is of particular interest within species such as *Vitis vinifera* L. where continuous variation in the level of isohydry has been observed among cultivars. With this objective, a 2 year experiment was conducted on the pseudo-F_1_ progeny from a cross between the two widespread cultivars Syrah and Grenache using a phenotyping platform coupled to a controlled-environment chamber. Potted plants of all the progeny were analysed for Ψ_M_, transpiration rate, and soil-to-leaf hydraulic conductance, under both well-watered and water deficit conditions. A high genetic variability was found for all the above traits. Four quantitative trait loci (QTLs) were detected for Ψ_M_ under water deficit conditions, and 28 other QTLs were detected for the different traits in either condition. Genetic variation in Ψ_M_ maintenance under water deficit weakly correlated with drought-induced reduction in transpiration rate in the progeny, and QTLs for both traits did not completely co-localize. This indicates that genetic variation in the control of Ψ_M_ under water deficit was not due simply to variation in transpiration sensitivity to soil drying. Possible origins of the diversity in (an)isohydric behaviours in grapevine are discussed on the basis of concurrent variations in soil-to-leaf hydraulic conductance and stomatal control of transpiration.

## Summary

This study on a grapevine mapping population shows that isohydric or anisohydric behaviour is under genetic control and is not simply controlled by transpiration response to soil drought.

## Introduction

Soil water deficit (WD) represents the main environmental constraint for growth in grapevine (*Vitis vinifera* L.) and grape production under Mediterranean conditions (Chaves *et al.*, 2010). When combined with high evaporative demand, soil drying may result in a dramatic decrease in water potential in plant tissues. This can lead to catastrophic effects such as cavitation in the xylem vessels (Zufferey *et al.*, 2011), cessation of water transport, stomatal closure, and carbon starvation, which threaten plant survival (McDowell *et al.*, 2008). Plants have evolved various adaptive processes limiting a dramatic decrease in leaf water potential in the daytime (Ψ_M_) under soil drought. As a result, contrasting controls of leaf water potential have been observed across species when submitted to similar soil WD conditions (Tardieu and Simonneau, 1998). So-called isohydric species, such as maize, efficiently maintain high Ψ_M_ when the soil dries, whereas anisohydric species, such as sunflower, cannot prevent Ψ_M_ from dropping (Tardieu *et al.*, 1996). In several species including grapevine (Prieto *et al.*, 2010), genetically variable efficiency of Ψ_M_ maintenance has been observed. Two widespread cultivars of grapevine, namely Grenache and Syrah, have been consistently described with different responses to soil WD. Grenache was shown to be near-isohydric, compared with Syrah which exhibited more anisohydric behaviour (Schultz, 2003; Soar *et al.*, 2006).

It has been proposed that the variation between isohydric and anisohydric behaviours mainly results from how stomatal pores at the leaf surface close under WD and control plant transpiration (Buckley, 2005). With regard to this, stomatal conductance to water vapour has been shown to decrease for milder soil WD in maize (isohydric) than in sunflower (anisohydric) (Tardieu and Simonneau, 1998). The higher stomatal sensitivity to WD in isohydric compared with anisohydric species could therefore be the cause of the more efficient maintenance of Ψ_M_. The drought-induced hormone abscisic acid, which triggers stomatal closure, was suspected to be at the origin of this difference (Tardieu and Davies, 1992; Borel *et al.*, 1997; Tardieu and Simonneau, 1998). However, the physiological basis of this proposal has never been elucidated. Additionally, it has been contested that (an)isohydric behaviour was stable for a given genotype but could vary seasonally (Franks *et al.*, 2007; Chaves, 2010).

Plant hydraulic conductance (i.e. a plant’s capacity to supply soil water to the leaves) participates in determining Ψ_M_ as it balances the impact of water losses. Variation in plant hydraulic conductance was therefore proposed to contribute, concurrently with stomatal regulation, to the control of Ψ_M_ under adverse conditions (Franks *et al.*, 2007; Pantin *et al.*, 2013). Accordingly, Ψ_M_ should remain high in those plants which could maintain high hydraulic conductance under soil drying. Conversely, vulnerability of the xylem pathway to embolism under drought most often causes a decrease in hydraulic conductance as observed in field-grown grapevine (Zufferey *et al.*, 2011), thereby lowering Ψ_M_ and resulting in anisohydric behaviour. This potential role of hydraulic conductance is supported by studies where different responses of hydraulic conductivity in roots (Vandeleur *et al.*, 2009) or petioles (Schultz, 2003) between grapevine cultivars under water stress have been related to their contrasted behaviours in terms of being isohydric or anisohydric.

The present study aims at elucidating whether and how variation in (an)isohydric behaviour is genetically controlled. This was addressed using a quantitative trait locus (QTL) mapping population consisting of the pseudo-F_1_ progeny obtained from a reciprocal cross between the two cultivars Syrah and Grenache. The range of genetic variation in Ψ_M_ observed in the progeny under controlled transpiring conditions was quantified with either a well-irrigated regime or moderate soil WD in pots. The genetic architecture underlying variation in Ψ_M_ within the progeny was then examined by looking for genomic regions associated with the control of this trait using a QTL approach. The transpiration rate and Ψ_M_ were characterized in parallel in all the progeny, and the genetic architecture of transpiration response to WD was similarly dissected. The physiological bases of isohydric versus anisohydric behaviours were discussed by matching correlations between traits to co-localizations between underlying QTLs.

## Materials and methods

### Plant material and treatments in the greenhouse

The plant material consisted of the pseudo-F_1_ progeny of 186 two-year-old genotypes obtained as the first generation from a reciprocal cross between the grapevine cultivars Syrah and Grenache (Adam-Blondon *et al.*, 2004).

In February 2010, 20 clones of each offspring and the parents were grafted on 110 Richter rootstock (*Vitis berlandieri*×*Vitis rupestris*) and then cultivated outside with ferti-irrigation in 9 litre (0.19 m diameter, 0.4 m high) individual pots containing a 30:70 (v/v) mixture of a loamy soil and organic compost. Six clones of each offspring and the parents were selected as replicates during a first experiment in 2012 (1128 two-year-old plants). Five other replicates (clones) were studied in 2013 (940 three-year-old plants).

In each year of the experiment, potted plants were transferred for budburst to a first greenhouse and grown there for 1 month. They were individually weighed and irrigated twice a week so as to maintain soil water content as non-limiting for growth (1.5g of water per g of dry soil; see Supplementary Fig. S1 available at *JXB* online). All inflorescences and branches were removed in order to ensure full growth of one leafy axis per plant.

**Fig. 1.**
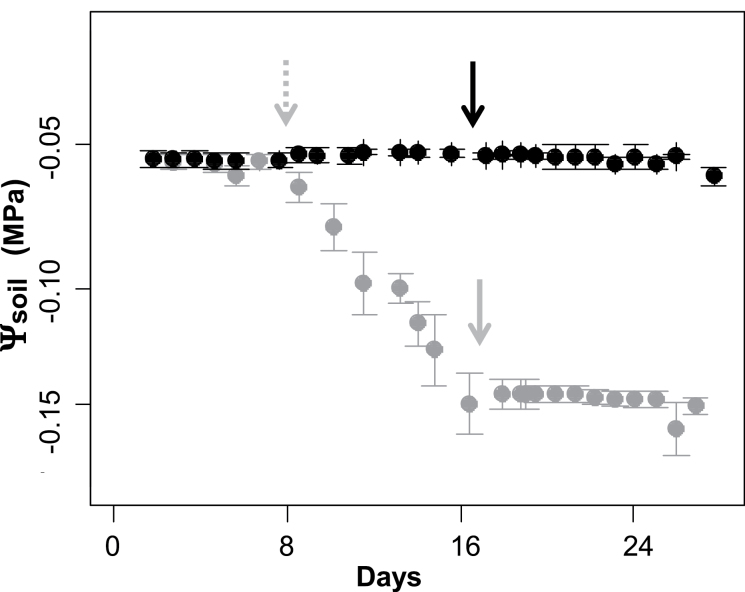
Evolution of soil water potential (Ψ_soil_) for potted grapevine plantlets cultivated in the greenhouse under both well-watered (WW, black circles) and water deficit (WD, grey circles) treatments (means and SEs for two replicates of the 186 Syrah×Grenache offspring and the two parents studied in 2013). The dashed arrow shows the beginning of water restriction. The black and grey arrows show the day of measurement of hydraulic traits, when Ψ_soil_ was stabilized under both scenarios.

The plants were then transferred into the PhenoArch phenotyping platform located in another greenhouse where water treatments were imposed. Briefly, PhenoArch is based on a LemnaTec automated system (LemnaTec, Wüerselen, Germany) hosted at Montpellier, France (M3P; https://www6.montpellier.inra.fr/m3p/). Within this platform, soil water contents (SWCs) in pots were maintained at target values by daily watering of each pot using watering stations made up of weighing terminals with 1g accuracy (ST-Ex, Bizerba, Balingen, Germany) and high-precision pump-watering stations (520U, Watson Marlow, Wilmington, MA, USA). For each genotype (186 offspring plus two parents), well-watered (WW) replicates (three clones in 2012 and two in 2013) were maintained at 1.50g of water per g of dry substrate. A moderate soil WD was imposed on three other replicates (clones) per genotype in each year, by restricting irrigation until the SWC gradually reached 1.05g g^–1^, and this level was maintained thereafter by daily watering for 1–3 d pending hydraulic measurements. Target weights used for daily irrigation were calculated for each pot as the sum of the desired amount of water corresponding to target SWC and individual tares which were determined during pot filling (empty pot, dry soil) including individual plant biomasses which were re-estimated twice a week by image analysis (see below). The target SWC for WD treatment was reached on average 7 d after restricting irrigation (Fig. 1) which was not long enough to alter the plant leaf area of WD plants compared with WW plants (see below).

In 2012, mean air temperature and vapour pressure deficit (VPD) in the platform greenhouse were 21.4 °C and 1.38 kPa during the night and 25.6 °C and 2.16 kPa during the day, and the photosynthetic photon flux density (PPFD) during the light period averaged 290 μmol m^–2^ s^–1^. In 2013, cloudier conditions resulted in lower values, with mean air temperature and VPD of 18.8 °C and 1.01 kPa during the night and 22.4 °C and 1.53 kPa during the day, with an average PPFD of 272 μmol m^–2^ s^–1^ during the light period.

### Image acquisition and analysis

Plant RGB (1280×960) images taken from two horizontal, orthogonal directions plus one top view were captured every 2 d for all plants during the night-time (3D Scanalyzer, LemnaTec, GmbH, Wüerselen, Germany). Images were analysed to separate plant regions from background and used for estimating the whole plant leaf area (LA) and fresh biomass (B_Plant_) for each plant. Calibration curves were constructed using multiple linear regression models based on processed images taken in three directions against measurements of LA and B_Plant_. The LA calibration curve was obtained with all the individually scanned leaves of 50 plants of different genotypes and sizes in 2012 and 2013. Calibration curves for B_Plant_ were obtained by weighing all progeny plants in 2012 and 625 in 2013 at harvest. Root mean square error of prediction (RMSE) was 0.031 m^2^ for LA and 10.7g for B_Plant_ (Supplementary Fig. S2 at *JXB* online).

### Experimental design

The experimental design consisted of parallel blocks in the phenotyping platform, and one clone of each genotype was randomly positioned within each block. Plant development was followed twice a week by counting the number of unfolded leaves. To eliminate uncontrolled plant size effects on water relations (Ambrose *et al.*, 2009), hydraulic traits were measured at a similar mean developmental stage for the total progeny except for two replicates of WW plants in 2012 which were measured at slightly earlier stages therefore resulting in smaller plants (LA=0.148±0.046 m^2^ as a mean for these two replicates over the progeny) compared with all other replicates and treatments for which mean plant sizes (±SEs) were similar (LA=0.212±0.065 m^2^ for WD plants in 2012; LA=0.242±0.092 m^2^ for WW in 2013; and LA=0.238±0.067 m^2^ for WD in 2013).

### Measurements of water relations in a controlled-environment chamber

Water potential and transpiration rate were measured under controlled transpiring conditions while plants were taken off the platform and placed in a controlled-environment chamber. Plant density in the chamber was reduced by one-third in 2013 compared with 2012 in order to facilitate sampling of exposed leaves. Plants were first submitted to a dark period with similar timing to that in the greenhouse (for ~12h). Lights were then switched on for a minimum of 3h before water potential measurements were performed. Air temperature and relative humidity (RH) were measured every 30 s (HMP35A probe, Oy, Helsinki, Finland) and the temperature was set to an average of 27 °C during the day (20 °C during the night). VPD was controlled by manipulating RH and was maintained at 2±0.2 kPa during the light period. Light was provided in the chamber by a bank of sodium lamps that maintained the PPFD at ~480 μmol m^–2^ s^–1^ at the level where leaves were sampled.

Before being placed in the chamber, pots were bagged to prevent evaporation from the soil. Each pot was then weighed with 0.1g accuracy (Sartorius balance, IB 34 EDEP, Gottingen, Germany) at the beginning of the light period and after a minimum of 6h under constant light, temperature, and VPD conditions. Weight losses over the 6h period were used to calculate average daytime transpiration rates on a whole plant (Tr) and a leaf area basis (TrS=Tr/LA, hereafter called the specific transpiration rate). Weight losses and LA were corrected by the weight and surface area of the leaf that was sampled for water potential measurement.

For each plant, SWC (g water g^–1^ dry soil) was calculated using the weight (Wt_total_) measured just before entering the controlled-environment chamber, corrected by the estimated plant biomass (B_plant_), as:

$$\text{SWC}=\left({\text{Wt}}_{\text{total}}–{\text{B}}_{\text{plant}}–{\text{Wt}}_{\text{pot}}–{\text{Wt}}_{\text{dry soil}}\right)/{\text{Wt}}_{\text{dry soil}}$$

with Wt_pot_ the weight of the empty pot and Wt_dry soil_ the weight of dry soil determined when plants were potted (2250g on average). Error in the SWC was reduced to <0.8% (errors were ±10.7g for B_plant_ and ±1g for Wt_dry soil_, Wt_pot_, and Wt_total_, resulting in <0.1% error for the denominator and <0.7% error for the numerator).

The soil water potential (Ψ_soil_) was then calculated for each plant using a calibration curve (Van Genuchten *et al*., 1980) previously established between SWC and Ψ_soil_ using pre-dawn leaf water potential determined under non-transpiring conditions on fully irrigated plants as a proxy for Ψ_soil_ (Supplementary Fig. S3 at *JXB* online):

$$\Psi_{\text{soil}}={\left(\frac{\text{SWC}}{{\text{SWC}}_{\text{sat}}}\right)}^{\left(\frac{-n}{n-1}-1\right)}{}^{\frac{\frac{1}{n}}{-\frac{\alpha }{10}}}$$

with SWC_sat_=2, *n*=1.468, and α=0.258. Using this calibration curve, soil water potential could be estimated from SWC with an RMSE of 0.06MPa (Supplementary Fig. S3 at *JXB* online). Pre-dawn leaf water potential was chosen as a proxy for Ψ_soil_ since it incorporates the influences of root distribution and distributed soil water potential in the pot on how the plant senses soil water availability. Further, calculation of water potential differences between transpiring leaves and soil (see below) was based on the same measurement technique.

The leaf water potential under transpiring conditions, as indicative of minimal daytime values (denoted Ψ_M_), was measured on the plants in the controlled-environment chamber between 3h and 4h after lights were switched on, with up to six Scholander pressure chambers (Soil Moisture Equipment Corp., Santa Barbara, CA, USA) which were cross-calibrated using a distributed, pressurized nitrogen source. Measurements were performed on fully expanded, well-irradiated leaves, generally on the eighth phytomer from the apex. The drop in water potential between soil and transpiring leaves was calculated as:

$$\Delta \Psi =\left(\Psi_{\text{soil}}–\Psi_{\text{M}}\right)$$

Whole-plant (K) and specific (KS) soil-to-leaf hydraulic conductance were then deduced using the conventional, evaporative flux method (Tsuda and Tyree, 2000) for the whole path from soil to transpiring leaves as:

K=Tr/ΔΨ

and

KS=TrS/ΔΨ

The stability of the transpiration rate over the 6h light period was verified in a preliminary experiment to ensure that Tr could be associated with the leaf water potential measured at any time during this period (Supplementary Fig. S4 at *JXB* online).

### Statistical analyses

All statistical analyses were performed with R packages (R Development Core Team, 2012). Natural logarithm transformation was applied when data distribution deviated from normality (Shapiro–Wilk test; Royston, 1995). Single effects of year, water scenario, and genotype were first tested on all traits. Effects of water scenario and interaction with genotype were then tested within each year (2012 and 2013) by using the following analysis of variance (ANOVA) model: *P*_*ij*_ *=*μ*+G*_*i*_ *+S*_*j*_ *+G*_*i*_ *S_*j*_ *+e*_*ij*_, where *P*_*ij*_ was the phenotypic value of genotype *i* in scenario *j*, μ the overall mean, *G*_*i*_ the effect of genotype *i*, *S*_*j*_ the effect of scenario *j*, and *e*_*ij*_ the residual error effect. The effect of year and interaction with genotype were tested within each scenario (WW and WD) using the same model, with *S*_*j*_ replaced by the effect of year *Y*_*j*_.

Further analyses were carried out on separate data sets (e.g. WD or WW, 2012 or 2013) to test for undesired effects (spatial position in the platform, date of measurement, and operator for leaf water potential measurement) by using one-way ANOVA models such as: *P*_*i*_=μ*+X*_*i*_ *+e*_*i*_, with *X*_*i*_ an undesired effect, and *e*_*i*_ the corresponding residual.

For each trait, the Best Linear Unbiased Predictors (BLUPs) of genetic values were then estimated for use in QTL detection. Models selected were those with the lowest Bayesian Information Criterion, among several mixed models (Supplementary Table S1 at *JXB* online). The tested models always included a random genotypic effect, completed or not by fixed effects and interactions reported as significant in previous ANOVAs for each data set (water scenario×year, multiscenarios, multiyears, all years and scenarios). Pearson’s correlation coefficients between years or traits were calculated on BLUPs of the genetic value.

Variance estimates of the selected models were used to estimate the broad-sense heritability (H^2^) as:

$${\text{H}}^{\text{2}}=\frac{\sigma^{2}{}_{\text{G}}}{\left(\sigma^{2}{}_{\text{G}}+\frac{\sigma^{2}{}_{\text{R}}}{n}\right)}$$

where σ^2^_G_ is the genetic variance, σ^2^_R_ the residual variance, and *n* the number of replicates per genotype.

### QTL detection

QTL detection was performed on elementary data sets (one water scenario within 1 year) as well as on multiscenario data sets (both scenarios in 1 year) and multiyear data sets (both years for one scenario). It was also performed on the whole data set considering the 11 clones of each genotype (six in 2012 and five in 2013) as replicates.

A framework linkage map was constructed using 153 simple sequence repeat (SSR) markers (Huang *et al.*, 2012). QTL detection was performed on BLUPs with MapQTL 4.0 software (Van Ooijen and Maliepaard, 1996) using the consensus map which combined information from the two parents. Interval mapping was performed in combination with multiple QTL model mapping (Jansen, 1993; Jansen and Stam, 1994) as an equivalent of composite interval mapping. QTL significance was determined at the chromosome (*P*_Chr_) and the genome-wide levels (*P*_G_) by calculating thresholds for the logarithm of odds (LOD) score through 1000 permutations (Churchill and Doerge, 1994). QTLs were declared significant when detected at the whole-genome level (*P*_G_<0.05), or putative when only significant at the chromosome level (*P*_chr_<0.05) (Abiola *et al.*, 2003). The confidence interval for each QTL was calculated as the chromosome region where the LOD score was higher than the maximum LOD score of the QTL minus 1.

Additive and dominance effects for the QTLs were calculated as described by Segura *et al.* (2007):

$$A_{\text{S}}=\text{1}[(\mu_{ad}+\mu_{a}{}_{c})-(\mu_{bd}+\mu_{bc})]/\text{4}$$

$$A_{\text{G}}=\text{1}[(\mu_{ac}+\mu_{b}{}_{c})-(\mu_{ad}+\mu_{bd})]/\text{4}$$

$$D=\text{1}[(\mu_{ac}+\mu_{b}{}_{d})-(\mu_{bc}+\mu_{ad})]/\text{4}$$

with *A*_S_ the additive effect associated with Syrah alleles, *A*_G_ the additive effect associated with Grenache alleles, *D* the dominance effect, and μ_*bd*_, μ_*bc*_, μ_*ac*_, and μ_*ad*_ the phenotypic means corresponding to the four possible combinations with *a* and *b* the alleles of Syrah, and *c* and *d* the alleles of Grenache.

## Results

### Mean effects of water treatments

All plants which were characterized in a controlled-environment chamber reached the desired soil water potential corresponding to either WW or WD treatment with high reproducibility between plants (the SE on Ψ_soil_ was <0.0014MPa and 0.014MPa for WW and WD plants, respectively) and years (mean Ψ_soil_ for all WD plants averaged –0.154±0.014MPa in 2012 and –0.151±0.005MPa in 2013).

**Fig. 2.**
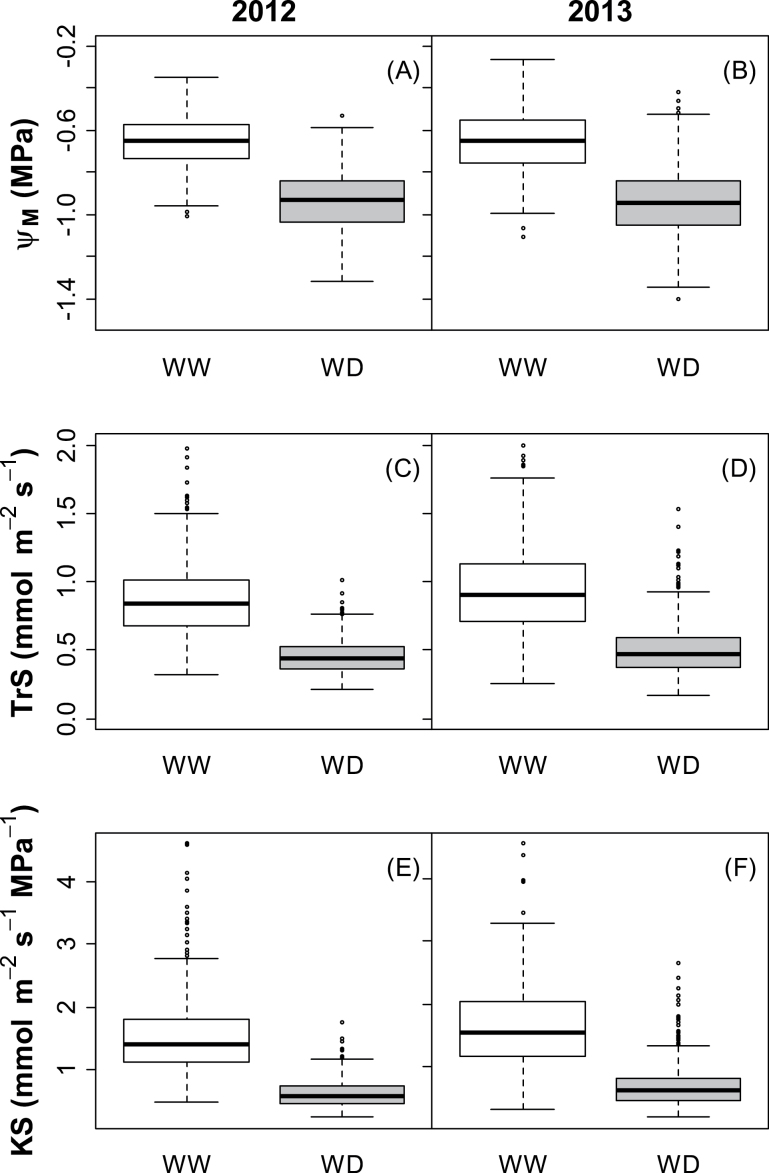
Boxplots of hydraulic traits measured on each plant of the Syrah×Grenache mapping population in 2012 (A, C, E) and 2013 (B, D, F), under well-watered (WW, white boxes) and water deficit (WD, grey boxes) treatments. See Table 1 for abbreviations (*n*=564 in all conditions except WW in 2012, where *n*=376).

WD treatment resulted in a highly significant decrease of all studied traits, with highly repeatable mean effects on plants between years (Table 1, Fig. 2). Mean Ψ_M_ for all measured plants was substantially reduced from –0.66MPa under WW conditions to –0.94MPa under WD treatment (Table 1, Fig. 2). Specific transpiration rate (TrS) and specific hydraulic conductance (KS) were slightly but significantly higher in 2013 compared with 2012. In spite of this difference, reduction induced by WD compared with WW conditions was of similar magnitude between years for mean KS, decreasing down to 43%, and mean TrS, which decreased down to 55% (Table 1, Fig.2).

**Table 1.** Effects of treatment (WW, well-watered; WD, water deficit), genotype, and year on the main hydraulic traits measured in two years on the Syrah×Grenache mapping population (two parents+186 offspring) in a controlled-environment chamber

Trait	Year	Effect of water treatment	Genetic and year effects					
		WW versus WD	WW			WD		
		*P*_S_	Mean	*P*_G_	*P*_Y_	Mean	*P*_G_	*P*_Y_
Ψ_M_ (MPa)	2012	****	–0.656±0.117	NS	NS	–0.934±0.14	****	NS
	2013	****	–0.658±0.142	NS		–0.938±0.16	****	
∆Ψ (MPa)	2012	****	0.599±0.117	NS	NS	0.78±0.138	****	NS
	2013	****	0.600±0.142	NS		0.787±0.16	****	
Tr (mmol s^–1^)	2012	***	0.11±0.05	***	***	0.08±0.03	***	***
	2013	***	0.21±0.11	***		0.1±0.05	***	
TrS (mmol m^–2^ s^–1^)	2012	****	0.87±0.26	****	***	0.46±0.13	****	***
	2013	****	0.95±0.33	*		0.51±0.19	****	
K (mmol s^–1^ MPa^–1^)	2012	***	0.19±0.11	**	***	0.11±0.04	***	***
	2013	***	0.37±0.24	**		0.14±0.09	***	
KS (mmol m^–2^ s^–1^ MPa^–1^)	2012	***	1.52±0.58	****	***	0.62±0.22	****	***
	2013	****	1.66±0.68	**		0.68±0.34	****	
			Mean			Genetic and year effects		
Red_Ψ_M_	2012	–	–0.280±0.106			*P*_G_,**; *P*_Y,_ NS		
(Ψ_WD_–Ψ_WW_)	2013		–0.280±0.132					
Red_TrS (TrS_WD_/TrS_WW_)	2012	–	0.544±0.125			*P*_G_,**; *P*_Y,_ NS		
	2013		0.558±0.163					
Red_KS	2012	–	0.422±0.127			*P*_G_,**; *P*_Y_, NS		
(KS_WD_/KS_WW_)	2013		0.441±0.176					

Means ±SD of 188 genotypic values determined on three clones of each genotype in each conditions, except WW in 2013 where two clones were measured.

Measured or derived traits were: Ψ_M_, daytime leaf water potential; ∆Ψ, difference between soil water potential and Ψ_M_; Tr, whole-plant transpiration rate; TrS, specific transpiration rate (on a per unit leaf area basis); K, whole-plant hydraulic conductance; KS, specific hydraulic conductance; Red_Ψ_M_, decrease in genotypic Ψ_M_ from WW to WD conditions (a single value for each genotype in each year); Red_TrS and Red_KS, the reduction ratio of TrS and KS, respectively. induced by WD relative to WW treatment (a single value for each genotype in each year).

The significance of water treatment (*P*_S_), genotype (*P*_G_), and year (*P*_Y_) effects is indicated as follows: *P≤0.08; **P≤0.05; ***P≤0.01; ****P≤ 0.001; NS, non-significant.

### Genetic variability within the progeny and comparison with parents

**Fig. 3.**
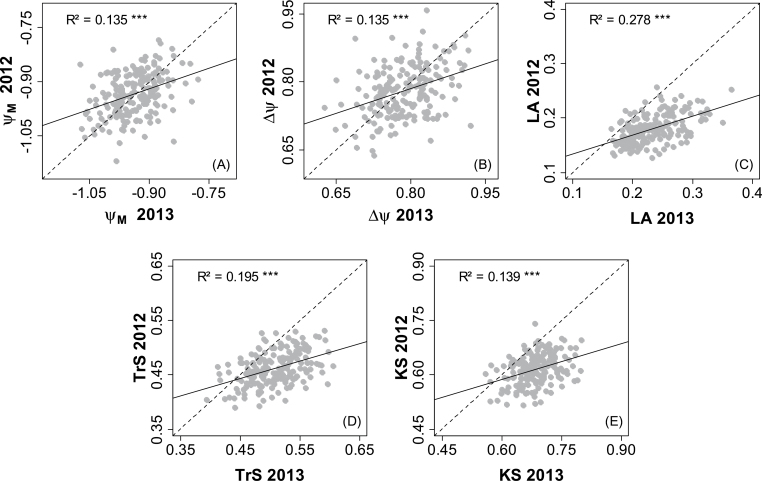
Comparison between 2012 and 2013 for mean genotypic values of hydraulics-related traits estimated under water deficit conditions for each offspring of the Syrah×Grenache mapping population. See Table 1 for abbreviations (Ψ_M_ in MPa, ∆Ψ in MPa, TrS in mmol m^–2^ s^–1^, KS in mmol m^–2^ s^–1^ MPa^–1^, LA in m^2^). *n*=188 for each year and each trait. Pearson’s determination coefficients are given, with *** indicating high statistical significance (*P*≤10^–9^). Regression lines are represented in black and bisecting lines in grey.

Mean genotypic values (BLUPs) were calculated using three replicates of each genotype submitted to the same treatment in each year (but only two for WW in 2013). Correlations between years were significant for each trait (as exemplified in Fig. 3 for WD) although with relatively low *R* ^2^ values (0.06–0.3), suggesting an interaction between genotypes and years.

**Fig. 4.**
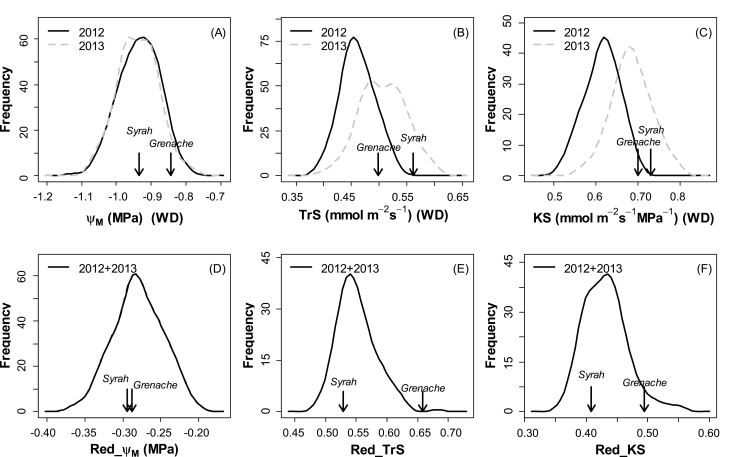
Distributions of mean genotypic values (Best Linear Unbiased Predictions; BLUPs) for hydraulic traits measured on the Syrah×Grenache mapping population. See Table 1 for abbreviations. For Ψ_M_ (A), TrS (B), and KS (C), distributions obtained under the water deficit treatment are presented for 2012 (black lines) and 2013 (grey lines) for the *n*=188 genotypes. For Red_Ψ_M_ (D), Red_TrS (E), and Red_KS (F), only one mean per genotype was obtained in each year, and the black line represents the distribution of the BLUP extracted from the single means of the two years of the experiment. Values for the parents are indicated with black arrows (only for 2012 in A, B, and C for the sake of clarity).

Parents were ranked in agreement with their previously reported behaviours under WD, with higher Ψ_M_ in the near-isohydric Grenache compared with the near-anisohydric Syrah (Fig. 4A). However, the difference between parents was rather weak compared with the range of variation observed among offspring.

ANOVA revealed highly significant effects of the genotype on Ψ_M_ under WD treatment but not under WW treatment, where mean genotypic values for Ψ_M_ were maintained between –0.78MPa and –0.6MPa (not shown). Segregation of Ψ_M_ under WD treatment within the progeny was therefore indicative of differences in (an)isohydric behaviours. Mean genotypic value of Ψ_M_ under WD ranged from –0.79MPa in 2012 (–0.78MPa in 2013), indicative of the most isohydric behaviour, to –1.12MPa in 2012 (–1.07MPa in 2013), indicative of the most anisohydric response (Fig. 4A). Genetic variability in (an)isohydric behaviours could also be appreciated through the range of variation in the reduction of Ψ_M_ induced by WD (Red_Ψ_M_), going from –0.17MPa for the most isohydric offspring to –0.40MPa for the most anisohydric one (Fig. 4D). However, Red_Ψ_M_ was obtained as the mean of only two values (one in each year; see the Materials and methods), while mean genotypic Ψ_M_ was calculated using three replicates in each year and was therefore preferred for assessing (an)isohydric behaviours.

A large and transgressive variability among offspring was also observed for both specific transpiration rate and specific hydraulic conductance (Fig. 4B, C). Mean, genotypic values under WD conditions ranged from 0.24 mmol m^–2^ s^–1^ to 0.76 mmol m^–2^ s^–1^ for TrS and from 0.28 mmol m^–2^ s^–1^ MPa^–1^ to 1 mmol m^–2^ s^–1^ MPa^–1^ for KS.

The response of the transpiration rate to WD was analysed by calculating for each genotype the reduction ratio of the specific transpiration rate induced by WD relative to WW conditions (denoted Red_TrS). Mean Red_TrS for 2012 and 2013 indicated that TrS was maintained at 66% of TrS observed under WW conditions in Grenache whereas it decreased to 53% in Syrah (Fig. 4E). This surprisingly disagreed with most studies describing Grenache as more water conservative under drought than Syrah. However, in spite of severe down-regulation of transpiration under WD in Syrah, TrS remained quite high (e.g. 0.56 mmol m^–2^ s^–1^ in 2012) compared with Grenache which was less affected by WD but exhibited a limited transpiration rate (0.50 mmol m^–2^ s^–1^ in 2012; Fig 4B). Reduction of the transpiration rate under WD also exhibited a wide range of variation among offspring far beyond the values reported for the parents (Fig. 4E), with a significant genotypic effect (Table 1). Interestingly, the genetic variability observed for the reduction of hydraulic conductance induced by the WD (Red_KS; Fig. 4F) was of similar magnitude to Red_TrS, with also higher reduction in Syrah (lower Red_KS) than in Grenache.

### Correlations between traits (genotypic values) within the progeny

**Fig. 5.**
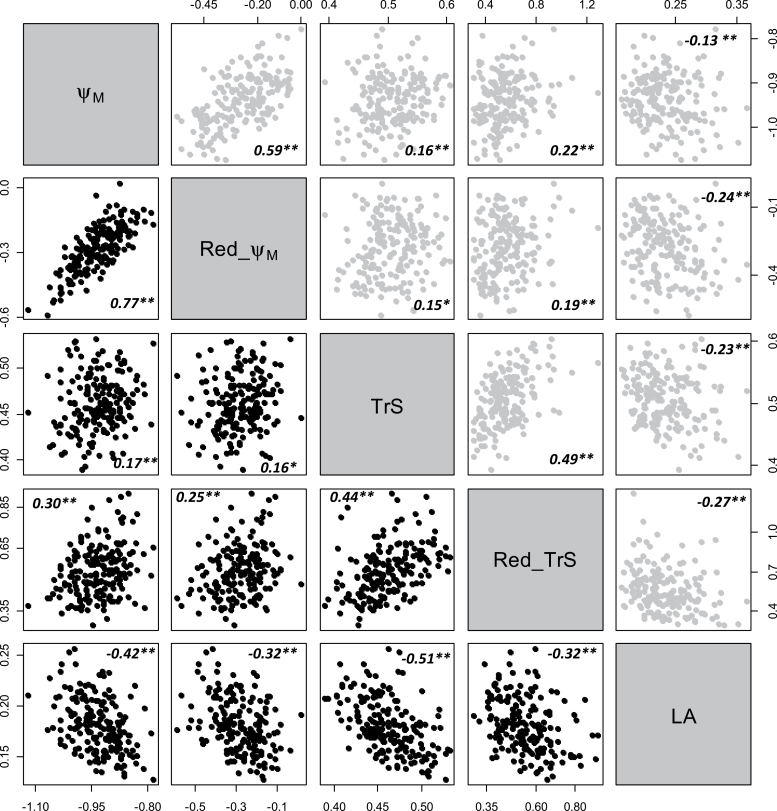
Binary relationships and Pearson’s correlation coefficients between hydraulic traits measured under the water deficit scenario on the Syrah×Grenache mapping population. Upper right: biplots between variables measured in 2013 (grey dots). Lower left: biplots between variables measured in 2012 (black dots). See Table 1 for abbreviations. Ψ_M_ in MPa, Red_Ψ_M_ in MPa, TrS in mmol m^–2^ s^–1^, Red_TrS dimensionless, LA in m^2^. *n*=188 for each biplot. Pearson’s correlation coefficients are indicated with their statistical significance as follows: *P≤0.01; **P≤0.001.

Relationships (Pearson’s correlation) between genotypic values (BLUPs) for all hydraulic-related traits were analysed in each year (Fig. 5). A significant effect of the genotype on LA was detected, indicating differences in vigour among offspring (Table 1). When normalized by plant leaf area, specific transpiration rate (TrS) measured under WD, as well as the reduction ratio Red_TrS, negatively correlated with LA (Fig. 5). This indicated a trend for the most vigorous genotypes to present the strongest reduction in transpiration rate and the lowest specific transpiration rate under WD.

It was examined whether genetic variation in Ψ_M_ correlated with genetic variation in transpiration rate under WD. Results were very similar in both years (Fig. 5). Correlation was weakly significant between mean genotypic values of Ψ_M_ and TrS and more significant between Ψ_M_ and the reduction ratio of specific transpiration (Red_TrS) calculated for each genotype. These correlations were positive, indicating that overall, genotypes with strongly reduced TrS under WD (low Red_TrS) were those with the lowest (most negative) leaf water potential. A loose, but significant, negative correlation was also observed between Ψ_M_ under soil WD and LA, suggesting that more vigorous genotypes exhibited lower leaf water potential under WD in spite of stronger reduction in specific transpiration as indicated by the negative correlation between LA and Red_TrS (Fig. 5).

### Mixed models and heritability

The broad-sense heritability (H^2^) of hydraulic traits ranged from 0 to 0.66 under the WW scenario and increased for each trait under the WD scenario up to 0.43–0.68 (Table 2). This indicated that soil drying amplified the genetic variability in hydraulic responses. For each trait, values calculated for both years were consistent. The whole-plant leaf area showed the highest H^2^ for both years. Analysis of the whole set of data combining the two years increased H^2^ values for all traits (Table 2), suggesting a higher power for QTL detection.

**Table 2.** Broad sense heritability (H^2^) of the main hydraulic traits (abbreviations as in Table 1) and leaf area (LA) measured on the Syrah×Grenache mapping population under the well-watered (WW) or water deficit (WD) scenario

Trait	Scenario	H^2^ (2012)	H^2^ (2013)	H^2^ (12+13)
Tr	All	–	0.652	0.717
	WD	0.544	0.6	0.616
	WW	0.476	0.494	0.519
TrS	All	0.677	–	0.788
	WD	0.546	0.572	0.676
	WW	0.548	0.442	0.662
K	All	–	0.55	0.618
	WD	0.432	0.549	0.54
	WW	0.459	0.395	0.4
KS	All	0.53	–	0.725
	WD	0.58	0.534	0.65
	WW	0.41	0.297	0.465
Ψ_M_	All	0.486	–	0.67
	WD	0.6	0.538	0.625
	WW	~0	0.232	~0
∆Ψ	All	–	–	0.66
	WD	0.59	0.53	0.612
	WW	~0	0.223	~0
LA	All	0.672	0.755	0.823
Red_Ψ_M_	WD – WW	–	–	0.344
Red_TrS	WD / WW	–	–	0.292
Red_KS	WD / WW	–	–	0.299

Heritability was calculated from genotypic and residual variances obtained by fitting mixed linear models, respectively, for the WW and WD scenarios, and for the multiscenario data sets (‘all’) when genotype×scenario interaction was significant.

### QTL analysis

**Fig. 6.**
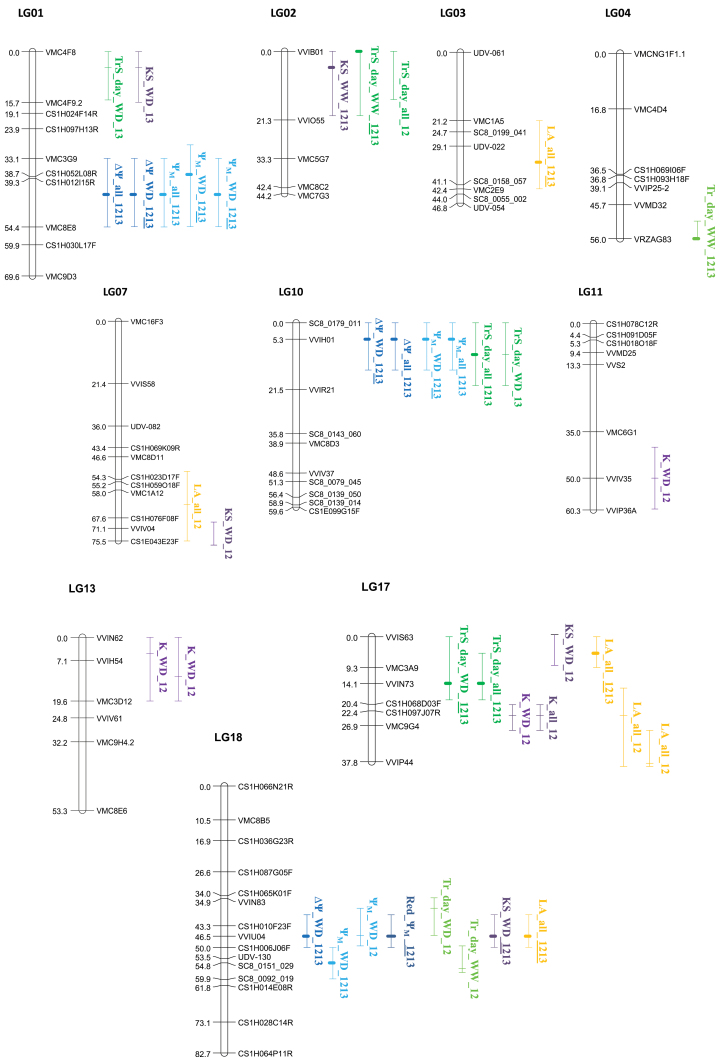
Localization of the most significant quantitative trait loci (QTLs) involved in the genetic determinism of the hydraulic-related traits in a Syrah×Grenache mapping population. Central marks on the vertical bars indicate the position L on each linkage group (LG01–LG18) of the consensus map where the maximum LOD score was observed (bold mark for multiyear QTLs and thin mark for 1-year QTLs). Vertical bars are for the LOD–1 confidence intervals around L. QTL names indicate the trait and the conditions (water treatment and years) retained for QTL analysis. QTLs were detected for: Ψ_M_, daytime leaf water potential; Tr and TrS whole-plant and specific transpiration rates, respectively; K and KS, whole-plant and specific hydraulic conductances, respectively; LA, whole-plant leaf area. QTLs represented here are mainly those detected on the multiyear data set (indicated as ‘1213’ in the QTL name). When a QTL was also detected in one specific year as highly significant, the corresponding year is underlined in the QTL name. Highly significant QTLs detected in 1-year but not in multiyear analysis are also represented and identified with the corresponding year ‘12’ or ‘13’ in the QTL name. The middle part of the QTL name indicates either ‘WD’, ‘WW’, or ‘all’ when a QTL was detected respectively using water deficit, well-watered, or all water treatment data sets. (This figure is available in colour at *JXB* online.)

QTL analysis of combined data for both years was carried out for each soil water condition. Twenty-two significant QTLs (*P*_G_<0.05) were detected by multiyear analysis (indicated as ‘1213’ in Table 3 and Fig. 6) of all hydraulics-related traits, although with some redundancy when QTLs were detected for those traits which were derived from others by calculation (such as for ∆Ψ or Red_Ψ_M_ as compared with Ψ_M_). One to four significant QTLs (*P*_G_<0.05) were identified per trait, each accounting for 9–20% of total variance. The QTLs were located on eight chromosomes, but many co-localized on four linkage groups (LGs) (four on LG01, five on LG10, three on LG17, and four on LG18).

**Table 3.** Significant quantitative trait loci (QTLs) detected on the consensus map of the Syrah×Grenache mapping population for the hydraulics-related traits (abbreviations as in Table 1) and leaf area (LA)Results of multiyear (‘12+13’) and single-year (‘12’ and ‘13’) analyses are presented for well-watered (WW) and water deficit (WD) scenarios, and for the multiscenario data sets (‘all’)

Trait	Year	LG	LOD	L (cM)	CI	%V	Effect
Tr WW	12	18	4.88	53.5	46.5–54.8	12.3	*A*_G,_ *D*
	12+13	**4**	**4.12**	**56**	**50.7–56**	**9.9**	***A***_**S**_
Tr WD	12	**18**	**4.26**	**34.9**	**31.6–43.3**	**10.2**	***A***_**S**_ **, *A***_**G**_
TrS all	12	**2**	**4.84**	**0**	**0–15**	**12.3**	***A***_**S**_ **, *A***_**G**_
	12+13	**10**	**4.23**	**10.3**	**0–20.3**	**12.6**	***A***_**S**_ **, *A***_**G**_
		**17**	**4.42**	**14.1**	**5–19.1**	**9.4**	***A***_**S**_ **, *A***_**G**_
TrS WW	12	2	4.8	5	0–20	13.3	*A*_S_, *A*_G_
	12+13	**2**	**4.32**	**0**	**0–20**	**10.8**	***A***_**S**_ **, *A***_**G**_
TrS WD	12	**17**	**4.6**	**5**	**0–14.1**	**12.8**	***D***
	13	**1**	**4.41**	**5**	**0–15**	**11.6**	***A***_**S**_
		**10**	**4.71**	**10.3**	**0–20.3**	**12.5**	***A***_**S**_ **, *A***_**G**_
	12+13	**17**	**5.09**	**14.1**	**0–19.1**	**10**	***A***_**S**_ **, *A***_**G**_
K WD	12	**11**	**4.13**	**50**	**40–60**	**8.2**	***A***_**S**_
		13	4.25	5	0–19.6	9.5	*A*_S_, *D*
		13	4.31	12.1	0–19.6	10.2	*A*_S_, *D*
		17	4.39	22.4	19.1–26.9	9.8	*A*_S_, *A*_G_
KS all	12	17	4.03	22.4	19.1–26.9	10.4	*A*_G_, *D*
KS WW	12+13	2	4.40	5	0–20	12.4	*A*_S_, *A*_G_
KS WD	12	7	5.42	75.5	67.6–75.5	10.5	*A*_G_
		**17**	**5.18**	**0**	**0–9.3**	**9.9**	***A***_**S**_ **, *D***
		**18**	**4.63**	**46.5**	**39.9–50**	**8.6**	***A***_**S**_ **, *A***_**G**_
	13	**1**	**4.17**	**5**	**0–15.7**	**12.3**	***A***_**S**_
	12+13	**18**	**4.69**	**46.5**	**39.9–50**	**10**	***A***_**S**_ **, *A***_**G**_
Ψ_M_ all	12+13	**1**	**4.62**	**44.3**	**33.1–54.4**	**12.7**	***A***_**S**_ **, *A***_**G**_
		**10**	**6.33**	**5.3**	**0–15.3**	**15.2**	***A***_**S**_
Ψ_M_ WD	12	**18**	**4.38**	**43.3**	**34.9–46.5**	**11**	***A***_**S**_ **, *A***_**G**_
	13	1	5.65	38.1	28.9–54.3	12.1	*A*_S_
		1	6.38	44.3	28.9–54.3	15.9	*A*_S_
		**10**	**5.07**	**5.3**	**0–15.3**	**12.2**	***A***_**S**_
	12+13	**1**	**5.02**	**38.1**	**28.9–54.3**	**12.1**	***A***_**S**_ **, *A***_**G**_
		**1**	**5.50**	**44.3**	**33.1–54.3**	**12.4**	***A***_**S**_ **, *A***_**G**_
		10	5.29	5.3	0–15.3	11.1	*A*_S_
		**18**	**5.32**	**54.8**	**50–59.9**	**11.1**	***A***_**S**_ **, *A***_**G**_
∆Ψ all	12+13	1	5.43	44.3	33.1–54.4	13	*A*_S_, *A*_G_
		10	6.16	5.3	0–15.3	14.8	*A*_S_
∆Ψ WD	13	1	5.61	38.1	28.9–54.3	12	*A*_S_
		1	6.35	44.3	28.9–54.3	15.8	*A*_S_
		**10**	**5.07**	**5.3**	**0–15.3**	**12.3**	***A***_**S**_
	12+13	1	5.79	44.3	33.1–54.3	12.9	*A*_S_, *A*_G_
		**10**	**5.69**	**5.3**	**0–15.3**	**12.1**	***A***_**S**_
		**18**	**5.78**	**46.5**	**39.5–50**	**12.1**	***A***_**S**_ **, *A***_**G**_
Red_Ψ_M_ WD–WW	12+13	**18**	**4.71**	**46.5**	**39.9–50**	**11.5**	***A***_**S**_ **, *A***_**G**_
LA all	12	**7**	**4.1**	**63**	**51.6–75.5**	**8.9**	***A***_**G**_ **, *D***
		**17**	**4.17**	**5**	**0–14.1**	**12.6**	***A***_**G**_ **, *D***
		17	4.25	22.4	14.1–37.8	9.5	*A*_G_, *D*
		**17**	**4.41**	**36.9**	**26.9–37.8**	**9.8**	***A***_**G**_ **, *D***
		**18**	**8.71**	**53.5**	**46.5–54.8**	**20.3**	***A***_**S**_ **, *A***_**G**_
	13	3	4.68	39.1	29.1–46.8	10.8	*A*_G_
		3	5.52	41.1	29.1–46.8	11.6	*A*_G_
		3	4.89	44	29.1–46.8	10.6	*A*_G_
		18	5.1	46.5	39.9–50	12.3	*A*_G_
	12+13	3	4.31	34.1	21.2–42.4	10.3	*A*_S_, *A*_G_
		**17**	**4.69**	**5**	**0–9.3**	**10.9**	***D***
		**18**	**8.42**	**46.5**	**39.9–50**	**19.5**	***A***_**S**_ **, *A***_**G**_

LG, linkage group; L, location of the QTL peak on the LG in cM; CI, confidence interval; %V, percentage of variance explained by the QTL; *A*_S_ and *A*_G_, the additive effects associated with Syrah and Grenache alleles, respectively; *D*, the global dominance effect.

The column entitled ‘effect’ indicates major effects of the considered locus involved in phenotype variation, satisfying the following condition: (|*A*s| or |*A*g| or |*D*|)/(|*A*s|+|*A*g|+|*D*|) >0.30.

QTLs in bold were significant in the mentioned year but also putative in the other year or in the multiyear analysis (see Supplementary Table S2 at *JXB* online for details).

Four significant QTLs were detected for Ψ_M_ under WD on LG1 (two QTLs), LG10, and LG18, and one of them co-localized on LG18 with the QTL detected for Red_Ψ_M_, both traits being indicative of (an)isohydric behaviour. One significant QTL was also detected for TrS under WD on LG17. No significant QTL was detected for the reduction of specific transpiration rate (Red_TrS) induced by the WD relative to the WW scenario.

Single-year and single-water treatment analyses revealed 32 significant QTLs (*P*_G_<0.05). On average, 1–5 significant QTLs were detected per trait, accounting for 8–20% of the total variance. Only one significant QTL was stable at the genome level in the two years (LA, on LG18). Nevertheless, for about half of the significant QTLs detected in the multiyear analysis, a significant QTL at the same locus was detected at least in one year, combined with a putative QTL (*P*_chr_<0.05) for the other year (Table 3). These redundant QTLs between years were not repeated in Fig. 6. In only two cases, significant QTLs specifically detected in one year under one water scenario were not reported as significant or putative in the multiyear analysis (Tr WW and KS WD in 2012) (Table 3, Fig. 6).

### Co-localizations of QTLs and clusters of interest

The different QTL analyses resulted in identification of genomic regions of particular interest, on LG01, LG02, LG10, LG17, and LG18 (Fig 6). The multiyear QTLs detected for Ψ_M_ tightly co-localized with QTLs for ∆Ψ (LG01, LG10, and LG18), suggesting that both traits carried similar information. This is consistent with the similar control of soil water potential which was imposed on all genotypes so that combining Ψ_soil_ with Ψ_M_ in the calculation of ∆Ψ did not introduce any substantial change in genetic architecture of these traits. A similar conclusion was obtained when analysing the difference in Ψ_M_ between WW and WD conditions (Red_Ψ_M_) since genetic variation was not detected under WW conditions in the multiyear analysis (Table 2). Regarding WD conditions, only one co-localization between QTLs of Ψ_M_ and specific transpiration rate (TrS) was detected on LG10. In contrast, more co-localizations were found between QTLs of soil-to-leaf hydraulic conductance (KS) and TrS on LG01, and LG17. This could be due to the way in which KS was derived from TrS. However, some specific QTLs were also detected for KS and TrS which did not co-localize.

Finally, to examine possible genetic links between plant size and hydraulic traits, QTL detection was also performed on LA. Although this trait varied slightly among replicates with the date of measurement and between years (with larger plants in 2013 compared with 2012, Fig. 3), QTLs were detected, suggesting that genetic differences were conserved across lots of plants (blocks and years). Two QTLs were detected in the main clusters of LG17 and LG18 (Fig. 6), where they co-localized with QTLs for specific transpiration rate or specific hydraulic conductance (Fig. 6).

Regarding the significant QTLs, the major effects were generally additive, originating from Shiraz (*A*_S_) and/or Grenache alleles (*A*_G_) and rarely associated with the dominance effect (*D*). The main QTL found for LA originated from both parents (Table 3).

## Discussion

### Phenotyping platforms facilitate the detection of genetic determinism of water relations

This is the first study where measurements of leaf water potential have been performed together with determination of the transpiration rate on all progeny from a cross. This work showed the benefit of using a phenotyping platform. By strictly controlling the individual soil water status through the daily weighing of pots, water shortage could be imposed on woody plants with good reproducibility between genotypes and years whatever the evaporative conditions and plant development. The platform also gave access to dynamic estimates of leaf area in a non-invasive way, which was essential in the determination of specific transpiration rates.

Furthermore, accurate control of atmospheric conditions in a growth chamber made it possible to characterize water relations of all the progeny for genetic analysis. Broad-sense heritability was quite high and consistent between years for most traits. For the first time, the genetic architecture of leaf water potential was analysed in transpiring plants under WD, and 32 QTLs involved in the control of water relations were detected. This offered a unique context to dissect the genetic origin of leaf water homeostasis and to shed new light on possible mechanisms causing differences between iso- and anisohydric behaviours.

### A genetic origin for (an)isohydric behaviours

Overall, the WD which was imposed in the present study on potted grapevine plantlets significantly decreased Ψ_M_ compared with WW conditions, but with a substantial range of variability within the progeny. As a major outcome, this variability in Ψ_M_ under WD appeared to be largely determined by the genotype. Genetic variability in Ψ_M_ under WW conditions was not detected. Therefore, genetic variability in Ψ_M_ under WD was indicative of the differences between more isohydric (high Ψ_M_ under WD) and more anisohydric (low Ψ_M_ under WD) behaviours. The wide range of genetic variation observed in the progeny compared with the parents suggested that several heterozygous QTLs were borne by the parental lines and segregated within the progeny. These conditions were favourable for the detection of QTLs governing the complex genetic determinism of the studied traits.

It should be noted that Ψ_M_ primarily depends on both soil WD and evaporative conditions, which were chosen as moderate in this study. The WD treatment corresponded to a moderate decrease in Ψ_soil_ (–0.15MPa for all the progeny) compared with non-irrigated field conditions where pre-dawn leaf water potential (a proxy for Ψ_soil_) frequently decreases down to –0.6MPa (Prieto *et al.*, 2010). Evaporative conditions in the controlled environment chamber were also chosen as intermediate by setting the VPD close to 2 kPa which has been shown to trigger intermediate stomatal responses in several species (Sinclair *et al.*, 2008; Gholipoor *et al.*, 2013; Ocheltree *et al.*, 2013), whereas a higher VPD induces severe stomatal closure (Monteith, 1995). In the experiments carried out here, the VPD was high enough to induce a genetically variable reduction in transpiration rate (Fig.2, Table 1) and thus, possibly, in Ψ_M_, but not too excessive to avoid severe limitations in the transpiration rate, which could have hindered the detection of genetic variation.

Genetic control of Ψ_M_ under stabilized evaporative and soil WD conditions was confirmed by the detection of several underlying QTLs on the consensus map. Four QTLs for Ψ_M_ under WD were detected in the multiyear analysis, each one explaining at least 11% of total variability. QTLs detected for Ψ_M_ under WD were most often highly significant in one year and putative in the other year. Difference between parental cultivars was modest and was dramatically transgressed within the progeny. This result is consistent with previous studies (Prieto *et al.*, 2010; Pou *et al.*, 2012), showing that the (an)isohydric reputations of Grenache and Syrah can be considered as overstated as compared with other cultivars. This also indicates possible genetic gains as regards leaf water homeostasis under drought.

### Transpiration rate under water deficit is genetically controlled but is not tightly correlated with variation in (an)isohydry

The transpiration rate was reduced by about a half by the mild WD conditions applied in the present study. Given that measurements were carried out in a strictly controlled environment, any change in transpiration rate could be ascribed to a change in stomatal conductance provided that the transpiration rate was expressed on a leaf area basis (i.e. by calculating the specific rate TrS). However, it should be noted that stomatal conductance may not be the only factor influencing transpiration and possibly involved in the transpiration response to WD. First, cuticular conductance should be considered. However, it was unlikely that the cuticle changed during the 7 days of soil drying preceding measurements. Similarly, boundary layer conductance in the controlled-environment chamber was mostly conserved between WW and WD conditions. In spite of strict control of atmospheric conditions in the chamber, TrS was slightly but significantly higher in 2013 compared with 2012 (regardless of water regime). This could be explained by the lower plant density in the growth chamber that was imposed in 2013 to facilitate leaf sampling. The reduction of plant density by about one-third in 2013 may have slightly increased the boundary layer conductance and probably resulted in more leaves exposed to direct light (which were selected for Ψ_M_ measurements in all cases). However, evaporative conditions were similar for WW and WD plants within each year. Eventually, within each year, only variations in leaf temperature induced by the reduction in transpiration rate under WD may have slightly distorted the relationship between specific transpiration rate and stomatal conductance. Stomatal closure in response to soil WD was therefore the primary cause of reduction in Trs induced by WD. A wide range of variation was found in the progeny, with slight reduction in transpiration rate for some genotypes (Red_TrS=0.78) down to stronger reduction for others (Red_TrS=0.42). A similar range of genetic variation in transpiration rate under WD was observed in scions grafted on a rootstock progeny (Marguerit *et al.*, 2012).

Variation in drought-induced stomatal closure, as approximated here by the reduction in transpiration rate, is thought to be at the origin of the contrasted behaviours between iso- and anisohydric plants (Buckley, 2005). This proposal, which has never been examined on a wide genetic scale, is challenged in this study by the absence of tight correlation under WD between Ψ_M_ (or Red_Ψ_M_) and speficic transpiration rate (or Red_TrS) within the progeny (Fig. 5). Even more puzzling, the corresponding correlations, although loose, were positive. This was not expected if a strong reduction in TrS (low Red_TrS and low TrS) associated with stomatal closure were to play a role in the maintenance of high Ψ_M_.

In other words, the way in which Ψ_M_ was decreased more or less severely by soil drying cannot be considered as simply determined by the range of variation in transpiration rate that was observed within the progeny. This is corroborated by the present results on QTL detection. Only one co-localization was found between a QTL detected for Ψ_M_ and specific transpiration rate (LG10), whereas other QTLs were specifically detected for Ψ_M_ on LG01 and LG18. This suggests, again, that the genetic controls of Ψ_M_ and stomatal regulation only partly share common determinisms and may also rely on independent mechanisms. It should be noted that heritability for reduction in transpiration rates (Red_TrS) was quite low in this study. Red_TrS was calculated as one single value for each genotype in each year and could not be determined with replications on individual plants which were exclusively characterized under either WW or WD treatment. This could explain why no QTL was detected for this trait. QTLs detected for specific transpiration were not systematically significant over the two years, as was the case in similar studies (Marguerit *et al.*, 2012). The most likely explanation is the high number of QTLs which are expected to control the transpiration rate in agreement with the large amount of genes identified as controlling stomatal differentiation (Barton, 2007) and functioning (Ward *et al.*, 2009). In such conditions, individual QTLs have weak effects and their detection becomes tricky.

Besides the role of stomata, it makes sense to question the contribution of hydraulic conductance in the control of leaf water homeostasis (Cochard *et al.*, 2002) since hydraulic conductance (from soil to leaves as considered in this study) determines the value of Ψ_M_ for a given value of transpiration rate and also because changes in hydraulic conductance may influence the stomatal control of transpiration (Pantin *et al.*, 2013). Within the Syrah×Grenache progeny, under WD, specific hydraulic conductance showed a high heritability and many QTLs were detected. Interestingly, although the calculation of KS was based on Ψ_M_ and therefore partly shared the same source of variation, only one QTL for KS co-localized with one of the QTLs detected for Ψ_M_ under WD (LG18). This suggests that, as for transpiration rate, genetic variability in hydraulic conductance may participate in the control of (an)isohydric behaviours but cannot fully account for them.

Over the whole grapevine progeny, WD reduced soil-to-leaf hydraulic conductance by about a half, but with contrasted responses within the progeny. This raises questions on the physiological origin of this genetic variability. Measurements in field conditions have already revealed rapid and severe impacts of soil drying on soil-to-leaf hydraulic conductance in grapevine cultivars, specifically in the daytime (Zufferey *et al.*, 2011). In the conditions used here, soil drying developed in ~1 week before measurements so that plant architecture (vessel diameter or leaf venation) was not greatly modified compared with WW plants during this short, terminal period of differential treatment. Moreover, WD treatment caused only a moderate decrease in water potential which was unlikely to have induced substantial cavitation in the xylem pathway. Therefore, the observed reduction in specific hydraulic conductance more probaly resulted from rapid changes in water transport capacity such as those already revealed in grapevine with the modification of aquaporin expression in roots (Vandeleur *et al.*, 2009) or leaves (Pou *et al.*, 2013).

The soil-to-root pathway which represents a major resistance to water flow (Tramontini *et al.*, 2013, in grapevine) is very sensitive to changes in soil water content (van Genuchten *et al*., 1980), and could have also contributed to the reduction in hydraulic conductance and to genetic variation within the progeny in the present study. However, the same rootstock (110R) was used for all offspring. Genetic differences originating in leaves rather than roots are more consistent with a variation in scions as involved in this study and with current knowledge on the effects of drought-induced signals such as abscisic acid on aquaporin activity in the sheaths of leaf veins (Shatil-Cohen *et al.*, 2011). Specifically, changes in some aquaporin activity in leaves induced by water stress have been correlated with changes in leaf hydraulic conductance (Pou *et al.*, 2013). However, it should also be envisaged that genetic variation of the scion was possibly accompanied by differences in rootstock development and root water transport activity, which merits further studies.

## Conclusions

The genetic analysis of water relations in the Syrah×Grenache progeny revealed that reduction in the transpiration rate induced by soil drying was not the only factor to be considered for the maintenance of leaf water potential in the daytime. Determinism of (an)isohydry should be sought in deviations from the balance between water supply capacity (hydraulic conductance) and stomatal control of transpiration. From a physiological point of view, this suggestion is consistent with the proposal that diversity in (an)isohydric behaviours originates in mechanisms uncoupling stomata from direct hydraulic control (Tardieu and Simonneau, 1998). From a genetic point of view, variability in (an)isohydric behaviour most probably rests on QTLs detected for the specific transpiration rate but not for specific hydraulic conductance (such as on LG10) and vice versa (LG13 and LG18). The present study also revealed some genetic determinism of Ψ_M_ variation under WD that appeared to be independent on both specific transpiration rate and specific hydraulic conductance (LG01). Recombining or selecting allelic variations in these QTLs appears to be a promising way to build cultivars with varying (an)isohydric behaviours.

## Supplementary data

Supplementary data are available at *JXB* online.

Figure S1. Mean shoot growth response of the main axis of cv. Grenache plants to soil water content.

Figure S2. Comparison between measured and predicted plant biomass and leaf area.

Figure S3. Relationship between soil water content (SWC) and soil water potential (Ψ_soil_).

Figure S4. Evolution of hourly transpiration rate during a 6h period after switching on the lights in a controlled-environment chamber and relationship between the transpiration rate calculated over the whole 6h light period and the 2h period when leaf water potential was determined.

Table S1. Mixed models selected for the extraction of BLUPs of genetic values.

Table S2. Putative quantitative trait loci (QTLs) (*P*_Chr_<0.05) detected on the consensus map of the Syrah×Grenache progeny for the hydraulics-related traits and leaf area measured in the Phenotyping Platform PhenoArch.

Supplementary Data
