# Supplementary material for: Genetic variation in a grapevine progeny (Vitis vinifera L. cvs Grenache×Syrah) reveals inconsistencies between maintenance of daytime leaf water potential and response of transpiration rate under drought
Source: J Exp Bot. 2014 Jun 13;65(21):6205–18. doi: 10.1093/jxb/eru228 (PMC4223985; doi:10.1093/jxb/eru228)
Supplement: Supplementary Data [file supp_65_21_6205__index.html]

Genetic variation in a grapevine progeny (Vitis vinifera L. cvs Grenache×Syrah) reveals inconsistencies between maintenance of daytime leaf water potential and response of transpiration rate under drought — Supplementary Data 

# Genetic variation in a grapevine progeny (*Vitis vinifera* L. cvs Grenache×Syrah) reveals inconsistencies between maintenance of daytime leaf water potential and response of transpiration rate under drought

## Supplementary Data

Data files

**Files in this Data Supplement:**

- Supplementary Data - Supplementary Data
