# Supplementary material for: Genetic variation in a grapevine progeny (Vitis vinifera L. cvs Grenache×Syrah) reveals inconsistencies between maintenance of daytime leaf water potential and response of transpiration rate under drought
Source: J Exp Bot. 2014 Jun 13;65(21):6205–18. doi: 10.1093/jxb/eru228 (PMC4223985; doi:10.1093/jxb/eru228)
Supplement: Supplementary Data [file supp_eru228_Coupel_Ledru_proof_supplement_revised_AU.pdf]

Genetic variation in a grapevine progeny (*Vitis vinifera* L. cvs Grenache X Syrah) reveals inconsistencies between maintenance of daytime leaf water potential and response of transpiration rate under drought.

Aude Coupel-Ledru, Éric Lebon, Angélique Christophe, Agnès Doligez, Llorenç Cabrera-Bosquet, Philippe Péchier, Philippe Hamard, Patrice This, Thierry Simonneau

Supplementary data

Supplemental Figure S1.

Mean shoot growth of the main axis of cv. Grenache plants grown under four soil water content (SWC) levels (1.5, 1.3, 0.8 and 0.7 g H_20 g dry soil^{-1}) in order to test the SWC corresponding to non-limiting conditions for growth. Each bar represents the mean ± SD of 10 replicates grown during a period of 15 days in a preliminary study conducted in 2011 in the PhenoArch Phenotyping Platform.

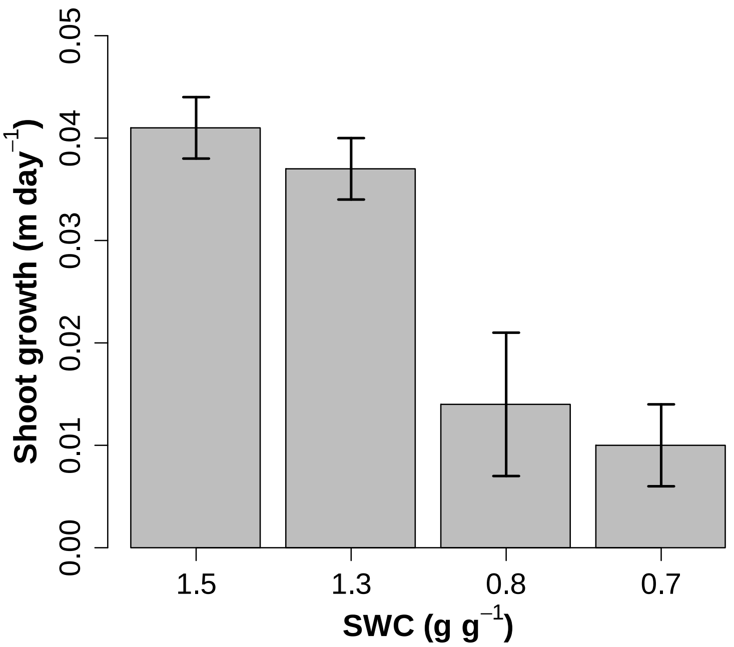

Supplemental Figure S2.

Projections of measured versus predicted plant biomass (g of fresh weight) (A) and leaf area (m²) (B). Estimated values were calculated from processed images using calibration curves. Calibration curves were obtained respectively by weighting all the plants of the whole progeny in 2012 and 625 plants in 2013 (A), and by individually scanning all the leaves of 50 plants of different sizes and genotypes in 2012 and 2013 (B). For biomass (A), calibration model was built using 2/3 of the data (n = 1192), and model was then validated on the other 1/3 of the data (n = 596). For leaf area (B), calibration model was built and validated using a 10 fold cross-validation. Mean root square errors of prediction (RMSE) were calculated on the validation sets.

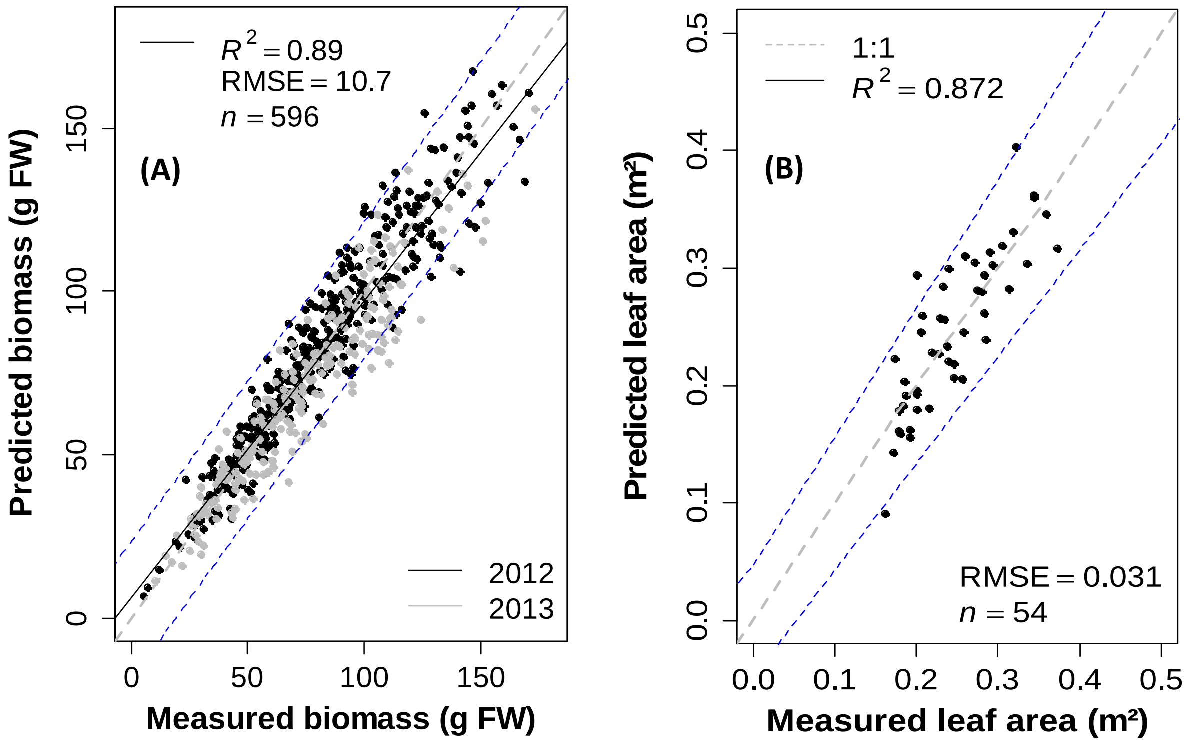

Supplemental Figure S3.

Relation between soil water content (SWC) and soil water potential (Ψ_{soil}). Measurements of predawn leaf water potential were used as a proxy for Ψ_{soil} and were carried out under non transpiring conditions on fully irrigated plants. Data were collected on randomly chosen genotypes within the Syrah X Grenache progeny (*n* = 90), together with maize plants cultivated in the same soil and conditions (*n* = 185). The calibration model was built using 2/3 of the data, and the model was then validated on the other 1/3 of the data. There was no significant difference between adjustments obtained for both species, so that the final adjustment was calculated using all the data. Mean root square error of prediction (RMSE) was calculated on the validation set, limited to data between -0.5 MPa and 0 MPa to allow a better accuracy in the interval of interest for this study.

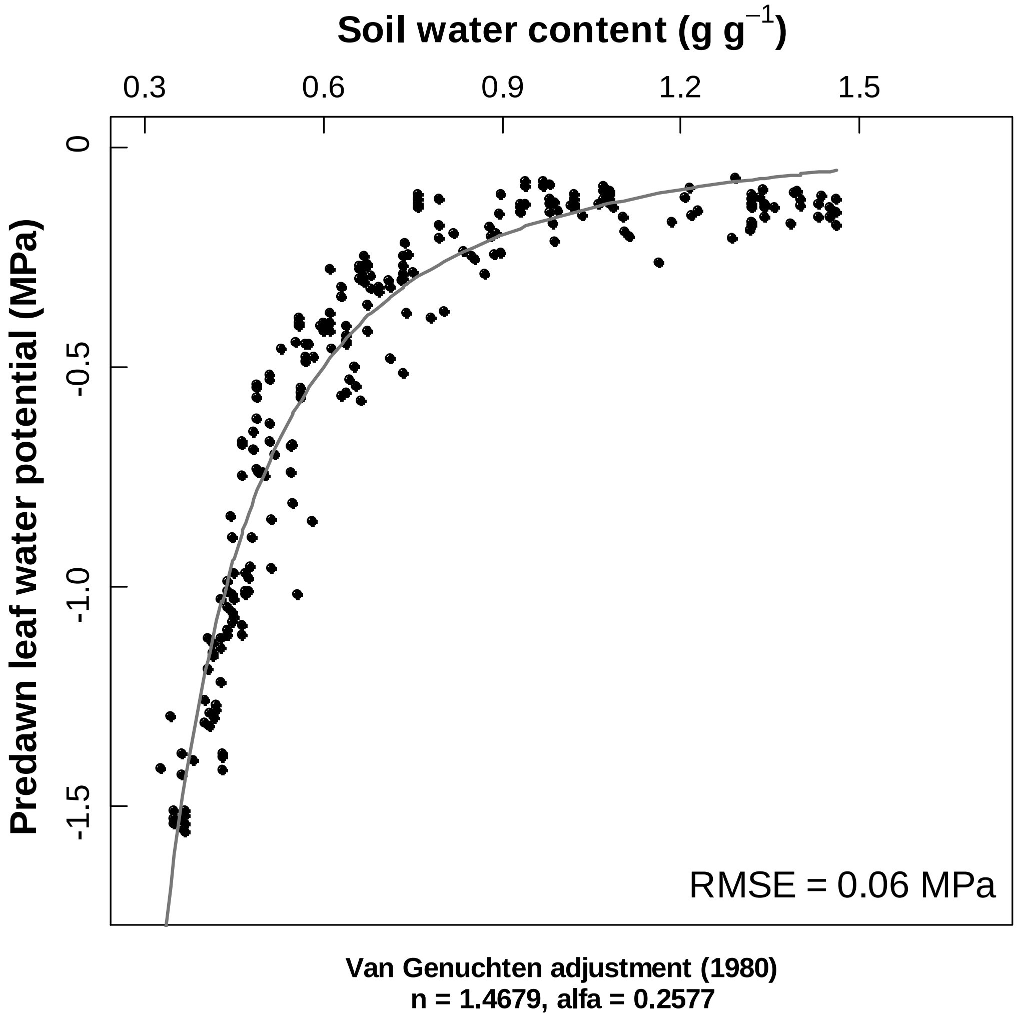

Van Genuchten adjustment (1980)
n = 1.4679, alfa = 0.2577

Supplemental Figure S4.

Evolution of hourly specific transpiration rate (TrS 1h) during the 6h period after switching on the lights in controlled-environment chamber and relation between specific transpiration rate calculated over the whole 6h light period (TrS 6h) and the 2h period (TrS 2h) during which water potential was measured (inset). Same protocol as described in the manuscript (Material and Methods) except that plants were weighed every 15 minutes. TrS 6h was calculated like in the manuscript as the total loss of weight per unit leaf area between the time when lights were switched and 6h later. In main plot, means and standard errors were calculated for n=7 plants of different genotypes randomly chosen within the Syrah X Grenache mapping population. For sake of comparison, hourly calculations were normalized by dividing by the 6h determination of transpiration rate. This ratio remained close to unity, indicating that transpiration rate was quite stable over the 6h period. In the inset, plants were either well watered (n=30, black points) or submitted to soil drying (n=16, grey points) and transpiration rate was calculated for the 2h period of time when water potential was measured. Points were distributed close to the bisecting line (dashed line) indicating that TrS 2h remained close to TrS 6h.

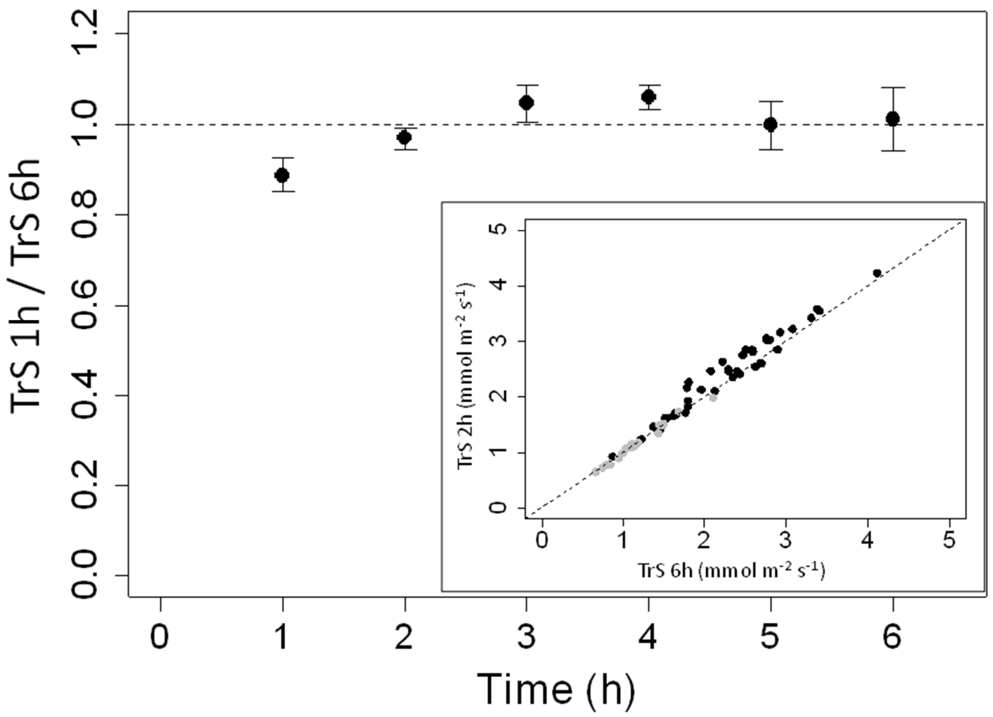

Supplemental Table S1.

Mixed-models selected for the extraction of BLUPs of genetic values. Models selected were those with the lowest Bayesian Information Criterion (BIC), among several mixed models.

Trait	Scenario	Year	Mixed-model
		2012	-
Tr	all	2013	$P_{ij} = \mu + G_i + S_j + G_i*S_j + e_{ij}$
		12+13	$P_{ijk} = \mu + G_i + Y_j + G_i*Y_j + S_k + e_{ijk}$
		2012	$P_{ijk} = \mu + G_i + D_j + X_k + e_{ijk}$
Tr	WW	2013	$P_{ij} = \mu + G_i + e_i$
		12+13	$P_{ij} = \mu + G_i + Y_j + G_i*Y_j + e_{ij}$
		2012	$P_{ijk} = \mu + G_i + D_j + e_{ijk}$
Tr	WD	2013	$P_{ij} = \mu + G_i + e_i$
		12+13	$P_{ij} = \mu + G_i + Y_j + G_i*Y_j + e_{ij}$
		2012	$P_{ij} = \mu + G_i + S_j + G_i*S_j + e_{ij}$
TrS	all	2013	-
		12+13	$P_{ijk} = \mu + G_i + S_j + G_i*S_j + Y_k + e_{ijk}$
		2012	$P_{ij} = \mu + G_i + Z_j + + e_{ij}$
TrS	WW	2013	$P_{ijk} = \mu + G_i + D_j + e_{ij}$
		12+13	$P_{ij} = \mu + G_i + Y_j + G_i*Y_j + e_{ij}$
		2012	$P_{ij} = \mu + G_i + e_i$
TrS	WD	2013	$P_{ijk} = \mu + G_i + D_j + e_{ij}$
		12+13	$P_{ij} = \mu + G_i + Y_j + G_i*Y_j + e_{ij}$
		2012	-
K	all	2013	$P_{ij} = \mu + G_i + S_j + G_i*S_j + e_{ij}$
		12+13	$P_{ijk} = \mu + G_i + S_j + G_i*S_j + Y_k + e_{ijk}$
		2012	$P_{ij} = \mu + G_i + X_j + e_{ij}$
K	WW	2013	$P_{ijk} = \mu + G_i + D_j + e_{ij}$
		12+13	$P_{ij} = \mu + G_i + Y_j + G_i*Y_j + e_{ij}$
		2012	$P_{ij} = \mu + G_i + e_i$
K	WD	2013	$P_{ijk} = \mu + G_i + D_j + e_{ij}$
		12+13	$P_{ij} = \mu + G_i + Y_j + G_i*Y_j + e_{ij}$
		2012	$P_{ij} = \mu + G_i + S_j + G_i*S_j + e_{ij}$
KS	all	2013	-
		12+13	$P_{ijk} = \mu + G_i + S_j + G_i*S_j + Y_k + e_{ijk}$
		2012	$P_{ijk} = \mu + G_i + X_j + Z_k + e_{ijk}$
KS	WW	2013	$P_{ij} = \mu + G_i + e_i$
		12+13	$P_{ij} = \mu + G_i + Y_j + G_i*Y_j + e_{ij}$
KS	WD	2012	$P_{ij} = \mu + G_i + e_i$
		2013	$P_{ij} = \mu + G_i + e_i$

		**12+13**	$P_{ij} = \mu + G_i + Y_j + G_i{*}Y_j + e_{ij}$
		**2012**	$P_{ij} = \mu + G_i + S_j + G_i{*}S_j + e_{ij}$
**Ψ_M**	**all**	**2013**	-
		**12+13**	$P_{ijk} = \mu + G_i + S_j + G_i{*}S_j + Y_k + e_{ijk}$
		**2012**	$P_{ij} = \mu + G_i + O_j + e_{ij}$
**Ψ_M**	**WW**	**2013**	$P_{ijk} = \mu + G_i + O_j + X_k + e_{ijk}$
		**12+13**	$P_{ij} = \mu + G_i + Y_j + G_i{*}Y_j + e_{ij}$
		**2012**	$P_{ij} = \mu + G_i + O_j + e_{ij}$
**Ψ_M**	**WD**	**2013**	$P_{ijk} = \mu + G_i + O_j + D_k + e_{ijk}$
		**12+13**	$P_{ij} = \mu + G_i + Y_j + G_i{*}Y_j + e_{ij}$
		**2012**	-
**$\Delta\Psi$**	**all**	**2013**	$P_{ijk} = \mu + G_i + O_j + D_k + e_{ijk}$
		**12+13**	$P_{ijk} = \mu + G_i + S_j + G_i{*}S_j + Y_k + e_{ijk}$
		**2012**	$P_{ij} = \mu + G_i + O_j + e_{ij}$
**$\Delta\Psi$**	**WW**	**2013**	$P_{ijk} = \mu + G_i + O_j + D_k + e_{ijk}$
		**12+13**	$P_{ij} = \mu + G_i + Y_j + G_i{*}Y_j + e_{ij}$
		**2012**	$P_{ij} = \mu + G_i + O_j + D_k + e_{ijk}$
**$\Delta\Psi$**	**WD**	**2013**	$P_{ijk} = \mu + G_i + O_j + D_k + e_{ijk}$
		**12+13**	$P_{ij} = \mu + G_i + Y_j + G_i{*}Y_j + e_{ij}$
		**2012**	$P_{ij} = \mu + G_i + X_j + e_{ij}$
**LA**	**all**	**2013**	$P_{ij} = \mu + G_i + D_j + e_{ij}$
		**12+13**	$P_{ijk} = \mu + G_i + S_j + G_i{*}S_j + Y_k + e_{ijk}$

P is the phenotypic value of genotype, *G* the random genotypic effect, *S* the fixed effect of water scenario, *Y* the fixed effect of year, *O* the fixed effect of operator for measurement of leaf water potential, *D* the fixed effect of date of measurement, *X* the fixed effect of spatial position on the *X* axis in the platform, *Z* the fixed effect of the spatial position on the *Z* axis in the platform, *e* the residual. Analyses were performed on the ‘multi-scenario’ data sets (‘all’) only when the interaction between genotype and scenario had a significant effect in previous ANOVA. In other cases, ‘-’ is indicated in the model column.

Supplemental Table S2.

Putative quantitative trait loci (QTLs) (P_{Chr} < 0.05) detected on the consensus map of the Syrah X Grenache progeny for the hydraulic-related traits and leaf area measured in the phenotyping platform PhenoArch.

Trait	Year	LG	LOD	L (cM)	CI	PEV%
**Tr WW**	12	4	2.66	56	50.7-56	6.5
	13	4	2.24	56	50.7-56	5.6
**Tr WD**	12+13	18	3.79	34.9	31.6-43.3	7.5
**TrS all**	12	10	2.24	10.3	0-20.3	6.7
		17	3.17	14.1	5-19.1	7.5
	12+13	2		0	0-15	8.6
**TrS WW**	12	2	3.89	0	0-20	11.8
**TrS WD**	12	10	1.61	10.3	0-20.3	4.9
		17	3.25	14.1	0-19.1	7.7
	13	17	2.07	5	0-14.1	4.5
		17	3.79	14.1	0-19.1	6.5
	12+13	10	2.88	10.3	0-20.3	10.3
**K WD**	12+13	11	1.24	50	40-60	3.6
**KS WD**	13	18	2.5	46.5	39.9-50	6.3
	12+13	1	2.47	5	0-15.7	7.5
**Ψ_M all**	12	1	1.27	44.3	33.1-54.4	3.8
		10	3.57	5.3	0-15.3	8.8
**Ψ_M WD**	12	10	2.41	5.3	0-15.3	5.8
		18	0.61	54.8	50-59.9	9.2
**Red_Ψ_M WD - WW**	12	18	3.29	39.9	31.2-50	9.4
	13	18	3.56	50	37-54	8.8
**$\Delta\Psi$ WD**	12	10	2.04	5.3	0-15.3	5.2
		18	3.67	46.5	39.5-50	9.4
	13	18	3.53	46.5	39.5-50	7.4
**LA all**	13	7	2.16	63	51.6-75.5	5.7
		17	3.17	5	0-14.1	10.3
		17	1.81	36.9	26.9-37.8	6.8
		18	4	53.5	46.5-54.8	10
	12+13	17	1.37	36.9	26.9-37.8	5.7

MQM method. Year, the year in which the trait was measured. LG, linkage group. L, position of the QTL peak on the LG in cM. CI, confidence interval. PEV, percentage of variance explained. Only putative QTLs detected at the locus where a significant QTL was detected on another year are represented.
